# Assessing feasibility and acceptability of study procedures: getting ready for implementation of national stroke guidelines in out-patient health care

**DOI:** 10.1186/s12913-015-1177-5

**Published:** 2015-11-23

**Authors:** Susanne Palmcrantz, Malin Tistad, Ann Catrine Eldh, Lotta Widén Holmqvist, Anna Ehrenberg, Göran Tomson, Christina B. Olsson, Lars Wallin

**Affiliations:** Division of Nursing, Department of Neurobiology, Care Sciences and Society, Karolinska Institutet, Stockholm, Sweden; Department of Clinical Sciences Danderyd Hospital, Karolinska Institutet, Stockholm, Sweden; School of Education, Health and Social Studies, Dalarna University, Falun, Sweden; Division of Physiotherapy, Department of Neurobiology, Care Sciences and Society, Karolinska Institutet, Stockholm, Sweden; Department of Clinical Neuroscience, Karolinska University Hospital, Stockholm, Sweden; Department of Physical Therapy, Karolinska University Hospital, Stockholm, Sweden; International Health Systems Research, Departments of Learning, Informatics, Management, Ethics and Public Health Sciences, Karolinska Institutet, Stockholm, Sweden; Mörby Academic Primary Healthcare Center, Stockholm County Council, Stockholm, Sweden

**Keywords:** Acceptability, Feasibility, Guidelines, Implementation, Stroke

## Abstract

**Background:**

Even though Swedish national guidelines for stroke care (SNGSC) have been accessible for nearly a decade access to stroke rehabilitation in out-patient health care vary considerably. In order to aid future interventions studies for implementation of SNGSC, this study assessed the feasibility and acceptability of study procedures including analysis of the context in out-patient health care settings.

**Methods:**

The feasibility and acceptability of recruitment, observations and interviews with managers, staff and patients were assessed, as well as the feasibility of surveying health care records.

**Results:**

To identify patients from the the hospitals was feasible but not from out-patient care where a need to relieve clinical staff of the recruitment process was identified. Assessing adherence to guidelines and standardized evaluations of patient outcomes through health care records was found to be feasible and suitable assessment tools to evaluate patient outcome were identified. Interviews were found to be a feasible and acceptable tool to survey the context of the health care setting.

**Conclusion:**

In this feasibility study a variety of qualitative and quantitative data collection procedures and measures were tested. The results indicate what can be used as a set of feasible and acceptable data collection procedures and suitable measures for studying implementation of stroke guidelines in an out-patient health care context.

**Electronic supplementary material:**

The online version of this article (doi:10.1186/s12913-015-1177-5) contains supplementary material, which is available to authorized users.

## Background

### Stroke care and rehabilitation

The Swedish National Guidelines for Stroke Care (SNGSC) targeted in this study are based on the principles of equal care, greatest support to those in greatest need and cost effectiveness, and provide a systematic review of current scientific knowledge for policy makers, managers and health practitioners [1, 2]. Thus, the SNGSC may facilitate the equal allocation of health care resources and to support a high standard of health care [3]. Nevertheless, a national survey of stroke care in Sweden identified geographical inequalities in access to stroke care [4]. Although the SNGSC states that home based rehabilitation is a top priority [3], in some regions less than 10 % received rehabilitation interventions in their home after stroke onset [5]. Moreover, the mean percentage of patients in Sweden that expressed unmet needs of rehabilitation during the first year after stroke was as high as 41 % and reached 53 % in certain regions [6]. These findings indicate the necessity of bridging the gap between evidence-based knowledge and practice, and the need to explore ways to successfully facilitate the implementation of the national guidelines in clinical out-patient settings.

### Implementing guidelines in clinical practice

Multiple strategies directed at health professionals, such as written information, education, and audit and feedback are often used to facilitate implementation of guidelines in the clinical context, however their results are generally moderate [7]. In England and Canada, with a health care organisation comparable to the Swedish, user involvement was used to support implementation of stroke guidelines. A Delphi process used to develop a consensus document for implementation and focus groups and interviews preceded the development of user-friendly information regarding interventions in clinical practice. Semi-structured interviews were used to assess the context where the guidelines, e.g. a complex pathway for stroke rehabilitation, were to be implemented [8]. Individuals within an organization can positively or negatively affect an implementation process [9] but it has been argued that the context in which the evidence is to be implemented plays a crucial role in implementation of evidence [10]. In the conceptual framework Promoting Action on Research Implementation in Health Services (PARIHS), context is included as a key factor comprising aspects such as organizational culture, evaluation of performance, and leadership [11, 12]. The extent to which leaders can facilitate change has been found to be related to their ability to tailor actions to fit the nature of the evidence and contextual conditions [13]. Sweden is structured in local authorities, comprising 20 regions and 290 municipalities responsible for providing health care and welfare for the residents [14]. The regional organization of health care may differ as the local and regional health care authorities are mandated to allocate resources and organize health care autonomously [14]. At the Swedish health care units, the senior managers are responsible for enabling provision of safe and cost effective health care of good quality [15]. Another commonality is that outpatient rehabilitation post stroke should be initiated through a referral, by the stroke unit at the hospital to out-patient units. So far, little is known about how such contextual factors impact the implementation of guidelines in health care. Thus, to capture the process of implementing guidelines within out-patient health care, aspects of context such as managers’ leadership, the use of evaluation to assess performance and the culture of the health care setting need to be studied. Other crucial factors to be targeted, recognized by the PARIHS framework, are the nature of the evidence and how the implementation of evidence is facilitated [9].

### Piloting

Implementation studies and other complex interventions should be preceded by pilot studies [16, 17] and as proposed by Feeley et al. [18], feasibility and acceptability with regard to means and measures in data collection and analysis need to be captured. Thus, prior to a proposed full-scale study on a leadership intervention supporting managers in implementing guidelines, such as the SNGSC, the aim of this study was to explore the feasibility and acceptability of study procedures in data collection including a survey of the context of the health care setting. In detail this paper reports on:

1. the feasibility of:
  recruiting units, managers and staff
  surveying health care contexts
  recruiting patients
  surveying rehabilitation interventions
2. the acceptability of data collection procedures, as perceived by managers, staff and patients.

In addition, a parallel paper reports on the results of piloting an intervention supporting the managers in the implementation of SNGSC.

## Methods

### Design

This paper reports on a feasibility study, including both qualitative and quantitative data collection and analysis.

### Setting

Five out-patient units were identified to assess the feasibility of surveying diverse health care settings: 2 units were operating in a geographical area within a southern urban region (with a population of 225 000 inhabitants) and 3 units were operating in a geographical area within a mid-Sweden rural region (with a population of 10 000 inhabitants). The provision of out-patient stroke rehabilitation after discharge was organized differently in these settings. The included rehabilitation units were a) part of a hospital organization (2 units), b) part of a district health care unit, c) part of the municipality health and welfare and d) a self-sufficient rehabilitation unit. Four units provided home-based rehabilitation only and 3 units provided both home- and clinic-based rehabilitation.

### Recruitment of units, managers, staff and patients

First, the senior managers at the identified units were contacted for informed consent. After approval, each unit was requested to include a senior manager and a front line manager. Moreover, occupational therapists and physiotherapists providing rehabilitation interventions for patients with stroke (the 2 latter hereafter referred to as “staff”) were contacted as this feasibility study focused on 3 recommendations in the SNGSC involving interventions made by occupational therapists and/or physiotherapists (Table 1). All managers and staff received written and verbal information about the study and gave their informed consent.

**Table 1:** The 3 recommendations in the SNGSC and assessment tools used to assess targeted functioning

Recommendation	Specification	Assessment tools
1)“Training with physiotherapist” [3]	“Rehabilitation interventions aimed at improving motor function, balance, walking ability and daily life activities (ADL)” [3].	• Berg Balance Scale [33] • Rivermead mobility index [34] • 6 min walk test [35] combined with the Borg RPE scale [36] • Barthel Index [30] • Katz Extended ADL Index [37] • Stroke Impact Scale [31, 38]
2) “Training in ADL in the home setting after discharge” [3]	“Training in ADL in the home setting after discharge, in case of limitations in ADL post stroke, limits the risk of an unfavorable outcome and improves the ability to perform ADL” [3]	• Barthel Index [30] • Katz Extended ADL Index [37] • Stroke Impact Scale [31, 38]
3) “Task specific training” [3]	“Task specific training aiming to increase activity performance in specified activities among individuals with impaired movement- related function” [3]	Impaired movement-related functioning is assessed in recommendations 1 and 2

**Fig. 1:**
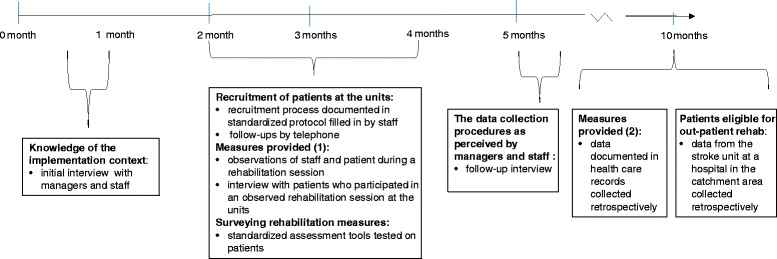
Data collection process. Provided in a separate file

Within a given time period of 2 months (Fig. 1), staff was instructed to initiate a consecutive recruitment of all patients who were referred to the units due to stroke-related needs for rehabilitation interventions within their first year after stroke onset. In close conjunction to their initial meeting with a patient, the staff was responsible for briefly presenting the study and for asking the patient if a data collector from the research team might contact him/her. Contact was made only by approval, and included verbal and written information. In case of limitations in communication due to aphasia a significant other was contacted and in case of language barriers registered interpreters were available. After consent, the patients were included in the study to be assessed with standardized measures. Among the included patients the staff identified patients who were planned for interventions by an occupational therapist and/or a physiotherapist and who could communicate in Swedish, the official language in Sweden. These patients were asked to participate in interviews and observations of provided rehabilitation interventions. The study was approved by the Regional Ethical Review Board in Stockholm (Regionala Etikprövningsnämnden i Stockholm).

### Data collection

The process applied to collecting data is presented in Fig. 1. Data collection followed a standard procedure and was performed by 2 registered physiotherapists/researchers (1 in the rural and 1 in the urban area) who communicated throughout the data collection process to assure consistency.

#### Surveying the health care contexts and participation in data collection procedures through interviews

All interviews were semi-structured and based on revised versions of previously tested interview guides [19]. To survey the health care context, from a manager and staff perspective, the PARIHS framework’s elements ‘leadership’, ‘culture’, ‘evaluation’, ‘evidence’ (in general and the SNGSC in particular), and ‘facilitation’ of knowledge implementation [11], were included in the interview guides used in a first interview with managers and staff. A second interview included questions addressing the feasibility and acceptability of the data collection procedures [18] used in this study (Fig. 1). Interview guides are provided in additional files (staff, Additional file 1 and Additional file 2, managers, Additional file 3).

To survey perceived conditions for implementation from a patient perspective, the interview guides for patients included questions related to their perceived functioning and the rehabilitation interventions they were receiving. Additional questions addressed the feasibility and acceptability of participating in the data collection including observations during a training session and in the research interview (Fig. 1). An interview guide is provided inadditional file (patients, Additional file 4).

#### Assessing the patient recruitment process using data from the stroke units at the hospitals

To assess the feasibility of the recruitment of patients, the proportion of patients referred to outpatient rehabilitation after stroke was surveyed by collecting data retrospectively, regarding patients discharged from the stroke units at the emergency hospitals in the 2 areas targeted in this feasibility study. The survey was based on data reported by the hospitals to Riksstroke, the Swedish Stroke Register [20], and included patients treated at the hospitals during the same period that patients at the out-patient units were included in the study to allow a comparison of the number of patients referred from the hospital to out-patient care after stroke with the number of patients identified at the units. The data consisted of: the number of patients treated and discharged from the stroke units at the hospitals; destination after discharge from the hospital and rehabilitation unit; and referrals made to rehabilitation units.

#### Surveying rehabilitation interventions using observations

Overt non participant observations were performed during rehabilitation sessions to survey interventions provided for patients in the context of the health care setting (Numbered 1, in Fig. 1). During these observations, an observation guide was used [21], also previously applied [19].

#### Surveying rehabilitation interventions through health care records at the rehabilitation units

Data from the patients’ health care records were retrieved to survey the rehabilitation interventions provided at the units. The data included rehabilitation interventions performed, the number and type of visits and duration of rehabilitation periods (Numbered 2, in Fig. 1).

#### Assessing patients’ functioning and disability with standardized assessment tools

Standardized assessment tools comprising aspects of the patients’ functioning and disability specified in the 3 recommendations in the SNGSC were tested (Fig. 1). The assessments were made face to face in the patients’ homes. While the aim was to test the procedures used in this feasibility study (rather than to evaluate patient outcome before and after an intervention), patients were assessed on one occasion using the tools outlined in Table 1. In addition, in order to to provide a more full-bodied picture of the patient’s functioning and disability, cognitive function was assessed with the screening tool Mini Mental Test examination [22]. The assessment tools were tested for floor and ceiling effects, the feasibility of the assessments, and acceptability as perceived by the patients.

### Data analysis

Descriptive statistics were used for quantitative data. The interviews were digitally recorded and transcribed verbatim. The observations were documented as field notes and reflections, all transcribed into a Word format [21]. Qualitative content analysis was used to analyze the observations and interviews with managers, staff and patients [23]. In the analysis of the first interviews with managers and staff, addressing the context of the health care setting, first, a naïve understanding was sought. Then, the texts were condensed, grouped and coded into subcategories and categories [23]. The subcategories that emerged after analysis of 5 interviews with managers were used to inform a matrix, including categories identified as corresponding to leadership, culture, evaluation, facilitation and evidence, in line with the PARIHS framework [12], and additionally the organizational structure and rehabilitation process. This matrix was then applied to the analysis of the remaining interviews with managers and staff [23]. In the analysis of the second interview addressing the feasibility and acceptability of the data collection procedures as proposed by Freeley et al. [18] a similar process was used. Here, the texts were condensed, grouped and coded into subcategories and categories corresponding to the feasibility and acceptability aspects of the recruitment and data collection. Trustworthiness was established by recurrent dialogues within the research team regarding the most valid understanding of the data and the rigor of the analysis [24].

The observations during rehabilitation sessions and interviews with patients were analyzed using another matrix, based on the 3 recommendations in the SNGSC (Table 1), also including the patient’s participation in the rehabilitation intervention [3]. The same matrix was also used to analyze the health care records where, in addition, documented use of standardized assessment tools was surveyed. The analyzed observations were compared to the notes in the health care records regarding the same rehabilitation session.

## Results

### Recruiting units, managers and staff

All senior and front-line managers approached gave their consent to include their unit in the study and the front-line managers did not report any difficulties related to the recruitment of staff members. Their numbers, years in position and sex is presented in Table 2.

**Table 2:** Managers and staff included in the study

Managers	*n*	Years in position < 5/≥ 5/>10	Women/Men
Senior managers	6	2/2/2	8/3
Front-line managers	5	1/3/1	
Staff members	*n*	Years in profession < 5/≥ 5/>10	
Occupational therapists	5	3/0/2	8/4
Physiotherapists	7	1/0/6	

### Surveying the health care contexts

The analyses of the initial interview with the managers showed that aspects related to leadership, culture and evaluation, facilitation and evidence had been captured, as well as aspects related to the organizational structure and the rehabilitation process. Findings are presented in Table 3.

**Table 3:** Findings related to the health care contexts based on interviews with managers

Framework	Interview findings
Leadership	Financial conditions were found to affect both senior and front-line managers’ ability to work with quality improvement.
	*“ ..well, you can do things as long as you keep your budget, but it’s very hard…..I find that sometimes the budget is an obstacle.”*
	*“So, the framework we are given is very governing. … to make ends meet in an organization, you need to do* this *kind of visit or* that *kind of treatment.”*
	The decision-making process at the units was described as two-tiered: decisions were made by the senior manager in consultation with the front line manager (at management level) or by the front-line manager in consultation with the staff (at clinical level).
	*“Yes, like education in this case… often we talk about it at the management board meetings … when there is something new… Should we send someone and what is the situation at the unit…then we have sent both occupational therapist and dietitian. In addition, I have had a few meetings with them, regarding what work material to order, what is reasonable… to go through with.”*
	The front-line managers were responsible for informing the staff about improvement initiatives determined at management level, and for leading the change of practice in collaboration with the staff.
	*“It may be X who initiates certain things, our senior manager too… Then it is up to the front line managers to proceed and, well, implement, in the units, I believe.”*
	Suggestions made by front-line managers and staff regarding changes in administrative and patient-related clinical routines then gained acceptance at management level where the final decisions were made.
	*“…if anyone has an idea, we have outpatient meetings where we can raise, perhaps, new…, if there are things related to the clinical routines that need to be changed”*
	*“We can decide quite a lot on our own. And I can raise a topic with the members of the management board and ask, ‘do you think we can do this this way, is it a good idea’?”*
Culture	While the staffs’ knowledge and support during change of practice were highly valued by the managers in some units, others found it hard to implement change due to a culture where the local staff was less inclined to change.
	*“Instead, it’s the staff members who sort of, well, see the possibilities and see when things don’t work.”*
	*“…we introduce something …then, it takes time. There … there are old buildings with an old culture, not easy to alter…”*
	The managers acknowledged their staff for their competence and ability to plan and execute rehabilitation interventions independently.
	*“And I find that staff members are tremendously good at pointing out and noticing, making suggestions.”*
Organizational structure	All units had experienced changes in their assignments concerning the provision of stroke-related rehabilitation interventions in out-patient care. within the last 6 months immediately prior to the start of the data collection
	For some units, the change entailed a new assignment while others had been assigned an expanded or reduced assignment.
	*“And when we formed this new organization now, by adding home based rehabilitation..”*
	*“And now the County Council claimed one full time employment, so it’s a big… well…”*
	The current assignments included interventions for patients with various diagnoses, including stroke.
	*“… the toughest change now is that……the stroke team’s assignment was expanded to a neuroteam, including other diagnoses in addition to stroke only.”*
Evaluation	In all units, the evaluations at unit level were focused on health care production (e.g. number of patients and types of visits) rather than outcomes in terms of patients’ functioning and disability.
	*“And the number of visits, the number of follow-ups… so now we are working on reporting once a month. To be able to follow up on how much health care we provide.”*
	*“…nothing standardized exists regarding that we do a good job.*
	Evaluation of patient outcomes was made on an individual level by the staff, after the rehabilitation intervention.
	*“We do follow-ups in the sense that… when you treat a patient you set a goal together with the patient. And then, you do an intervention that you agree on, together with the patient. And then, you perform the intervention that you agreed on and then, do the follow-up.”*
	No standardized procedures were used for evaluation of patient outcome.
	*“Well,….how we…sort of see that we really have a result. And this is where we sense that we don’t have a good, not yet, set, what we should use, really. So, it is a bit arbitrary.”*
Facilitation	The front-line managers described themselves as being responsible for creating conditions that would facilitate change in their units, either through their interaction with staff or by appointing a facilitator from among the staff.
	*“Well, really, …it’s my job, I would say. Changes, improvements, are what I do at work.”*
	*“…we do everything together with the staff to… I mean, they have the knowledge of how everything works.”*
	*“And try to allocate different areas of responsibility, so that everybody has something. Sometimes I pick a few, when I sense that the others are not able.”*
Evidence	The managers described various ways of staying informed about new scientific evidence available at the units. The staff was considered to be responsible for keeping themselves up-to-date.
	*“It’s the staff … really, you have two missions, you should do the job but you should also stay informed …I believe.”*
	According to the managers, the staff’s clinical experience and the identified needs of the patient were the primary approach used for guiding the clinical work.
	“..*. patients and significant others, first of all. But really, secondly it’s the improvement propositions from employees, I mean, staff.”*
	New national guidelines were not always perceived as clinically useful. According to the managers, the staff was already working according to national guidelines, or the guidelines were considered impossible to implement, due to lack of resources.
	*“..that this administrative process, in itself, regarding implementation of guidelines, works a lot faster in real life practice.”*
	*“evidence and guidelines are basically impossible to follow…there are no resources or personnel.”*
Rehabilitation process	Financial conditions directed the outline of rehabilitation interventions. Reimbursement mechanisms in 1 area directed the rehabilitation intervention (by price tagging different rehabilitation interventions), whereas allocation of resources for rehabilitation was guided by budget in the other area.
	*“How the rehabilitation interventions are outlined is partly a matter of resources, really, for us it’s entirely a question of assignment. What are we assigned to do, what do the administrators and politicians want, in the end.”*
	*“You can do things, as long as you stick to your budget.”*
	Standardized procedures to be used by staff during rehabilitation interventions were discussed by the front-line manager and staff. The standardization process was at different stages at the various units, and individual differences in the provision of the rehabilitation interventions was known, along with staff habits of using outdated routines.
	*“So there are large differences, that you need to identify, one may need to limit the interventions he/she provides and others may need to provide a bit more than before.”*
	*“…instead, you end up in…this is how we are used to do it.”*
	The content of the interventions, provided during the rehabilitation sessions, were directed by staff competence as well as the interaction between staff and patient, focusing on the patient’s needs.
	*“Partly, it’s up to each professional’s competence and wishes, what you want to do. Honestly, this affects how you treat each patient.”*
	*“…but….really, you need to see to, what needs do the patient have, this is what guides us, all the time.”*

In the initial interviews with staff, results from the interviews with managers relating to leadership, evaluation and the rehabilitation process were confirmed. Again, the financial conditions were found to direct rehabilitation interventions and evaluation focused on productivity. Moreover, the leadership decision-making process described by the managers was confirmed by the staff. In addition, the staff interviews confirmed that the content of the rehabilitation intervention was directed by the staff and patient’s needs and that the planning and execution of rehabilitation interventions differed between staff members. Multiple ways to stay informed about new evidence were described, but this was not done in a systematic manner. In terms of facilitation, staff members pointed to the necessity of involving staff members in changing practice in order to be successful in implementation.

### Recruiting patients at the units and the assessment of the recruitment process

Eighteen patients in the urban area and 6 patients in the rural area were approached by staff at the units. In 1 unit in the rural area no eligible patients were identified by staff during the time of inclusion. In the 2 remaining units, 1 patient at each unit were identified and included after given consent. To increase the number of included patients, another 4 patients were approached in an adjacent geographical area. Nine patients in the urban area and 2 in the rural area declined participation leaving 13 patients to be included in the study. Reasons for declining were: other dominating disease, the patient’s social situation, limitation in communication due to aphasia, or language barriers.

At the 2 local hospitals, one serving each area, patient data (over the course of the same 8 weeks as data was collected in the out-patient clinic) could be successfully retrieved retrospectively. During the recruitment period in the urban area, 64 patients were referred from the hospital to out-patient rehabilitation after stroke. Over the same period in the rural area 5 patients were referred from the hospital to out-patient rehabilitation after stroke.

In total, 13 patients were included and assessed by the data collectors (mean age: 73 years, SD 8, range 61–86; sex: 9 men, 4 women; time since stroke onset: 2 months, SD 2, range 1–6 months). All but 1 patient gave consent to access their health care records.

The staff reported that 7 of these 13 patients were planned for rehabilitation interventions according to the 3 recommendations in the SNGSC and were thus eligible for interviews and participation in the observations.

### Surveying rehabilitation interventions provided at the units

The notes from the observations were compared to the notes in the health care records regarding the same rehabilitation session. The observations made at the rehabilitation session were, in general, found to correspond to the notes in the health care records, in terms of documented activities performed during the sessions. However, contrary to the detailed observations, the health care records provided a summarized description of the interventions provided in the rehabilitation sessions. The observations generated information regarding the interaction between the patient and the staff, e.g. if and how the patient was involved in the choice of interventions provided by staff and the patient’s self-sufficiency in performing the exercises during the sessions. Moreover, the observations provided more detailed information regarding the type and intensity of the exercises and whether the interventions were task-oriented or not.

When all the notes in the health care records from the rehabilitation period were analyzed, a more comprehensive picture of the interventions provided emerged:

- All but 1 patient were found to be in need of and had received rehabilitation interventions according to the 3 recommendations in the SNGSC.
- Disability and task specific training according to the 3 recommendations in the SNGSC were documented in the health care records.
- All but 1 patient received all their rehabilitation interventions in the home and all but 1 patient received visits from both an occupational therapist (mean 5 visits, SD 3, range 2–10), and a physiotherapist (mean 5 visits, SD 5, range 0–17).
- The duration of the rehabilitation periods varied between 5 and 45 weeks (with a mean of 15 weeks).
- Use of standardized assessments tools to assess patient’s functioning, disability and outcome of the interventions according to the 3 recommendations were not found in the health care records, with the exception of 1 assessment of limitations in ADL.

All patients who participated in the interviews and were observed experienced disability of relevance to the 3 recommendations in the SNGSC. The interventions that the patients recalled and described were also found in the health care records. However, the health care records included information on additional interventions that were not described by the patients.

#### Assessing patients’ functioning and disability with standardized assessment tools

During the patient assessments performed by the data collectors, no adverse events were reported. All items included in the assessment tools could be completed in the patients’ homes, while the 6 min walking test was performed in the area surrounding the patients’ homes.

None of the included patients suffered from cognitive decline according to the Mini Mental Test examination (mean 26, SD 2) but impaired memory was self reported in the Stroke Impact Scale (mean 80, SD 13). Moreover, according to the results from the assessment with the Barthel Index, the included patients were all in the higher range (median 95, IQR 90–100), indicating a high proportion of patients who were independent in personal care activities and mobility. However, according to SIS the patients experienced limitations in personal care and domestic life (mean 78, SD 14) as well as in mobility (mean 79, SD 19) and in participation in meaningful activities (mean 68, SD 18). Thus, aspects of the patients’ functioning and disability not captured by the Mini Mental Test examination and the Barthel Index were captured by the Stroke Impact Scale. During the assessments with the Katz Extended ADL-Index, difficulties in responding to the questions regarding dependence in domestic activities or public transportation were identified for 5 patients who, by choice rather than disability, did not perform these activities.

### Data collection procedures as perceived by managers, staff and patients

All the managers, staff and patients took part in the semi-structured interviews. One manager did not wish to be digitally recorded, but agreed to notes being taken during the interview. No burden relating to the interviews was reported by the managers, staff or patients. All included patients gave their consent to being observed and assessed with the standardized tools for evaluation of patient outcome and completed their participation during these data collection procedures. Again, no perceived burden was reported by the patients.

According to the follow-up interviews, staff members had gained a good understanding of the patient recruitment process through the written information combined with the telephone conversation. However, in terms of the actual recruitment process, a more diverse picture evolved: while some found the recruitment process to be effortless, others had problems with e.g. finding words to describe the study to the patients. In addition, difficulties with developing good routines to execute the recruitment process were reported. Some found it hard to remember to inform the patients due to either a high work load or too few eligible patients. Also, the staff applied different interpretations of the inclusion criteria, and as a result they did not always inform all eligible patients about the study.

The staff’s perceived burden of participating in the recruitment of patients was affected by the working conditions at the units. In 1 unit, a high work load negatively affected staff members’ compliance with following the study procedures and participation was perceived as a burden. In other units, the staff’s participation was limited or could not be evaluated because few or no patients with stroke had been referred during the recruitment period.

## Discussion

In the following section, the feasibility of surveying health care contexts, in connection to implementation of guidelines such as the SNGSC, is discussed, as well as the feasibility and acceptability of the data collection procedures used in this study. The knowledge gained can be used in the design of full scale studies focusing on the implementation of guidelines in out-patient health care settings.

### Recruitment of managers, staff and patients

The units included represented rural and urban areas, different organizational structures, and with different financial conditions. A spread in sex and number of years in held positions was found among managers and staff as well as a spread in the characteristics of the patients included. Thus, results in this pilot are derived from a limited but presumably representative sample of Swedish out-patient health care settings where stroke related rehabilitation interventions are provided [14]. A limitation was the low number of inhabitants in the rural area which restricted the number of patients eligible for inclusion in the study. This finding indicates the need of surveying the demographics and the number of eligible patients in a standardized manner before inclusion.

The feasibility of recruiting patients from out-patient units can be questioned. When data regarding the number of discharged patients from the stroke units at the hospitals was compared to data regarding the number of patients approached at the out-patient units, results indicated that numerous patients were lost in the referral to the out-patient units and/or in the inclusion process at the units. It must be noted that not all patients discharged after stroke onset are in need of rehabilitation interventions. However, results from the Swedish national stroke register’s evaluation of the provision of home-base rehabilitation [5] and patients’ satisfaction with care [6] indicate that a substantial number of patients do not receive adequate rehabilitation interventions. These findings indicate that when designing a study regarding implementation of guidelines in out-patient care, the collaboration between the hospital and out-patient units need to be targeted, in order to minimize the potential risk of patients in need of rehabilitation interventions after discharge being lost in the referral process. This knowledge may be generalized to other diagnoses and different care trajectories. Overall, a crucial issue in implementation studies is how to optimize the identification of patients that will benefit from the recommended clinical interventions [17].

In the present study, the identification of eligible patients discharged to their homes and to rehabilitation was feasible when data was provided from the hospital. Another advantage of using this procedure is that eligible patients may be identified without burdening health practitioners. Notably, the recruitment process was found not to be acceptable due to the perceived burden, identified by the staff in 1 unit. These findings indicate a need to relieve the staff from of the responsibility of identifying, approaching, and informing the patients. Moreover, the fact that staff members at the units used different interpretations of the inclusion criteria illustrates the need to only use data collectors with full knowledge of inclusion and exclusion criteria for identifying and approaching patients. These results are in line with findings in a systematic review where issues related to the clinicians’ participation in the studies were reported. The review concludes that the patient-related recruitment process should be piloted, that demands on the clinicians should be minimized and that their participation needs to be supported by dedicated research staff [25].

### Surveying out-patient health care settings

Interviews with managers and staff were considered feasible and generated valuable knowledge regarding the context of the out-patient health care setting. However, interviews with front-line managers may be considered sufficient to gain knowledge of the context as the interviews with staff provided no additional information. As time is often limited for study participants, the necessity of collecting affirmative data needs to be balanced with the burden that participants may experience [18].

Given the leadership focus, the PARISH framework that contains a specified leadership element was thought to be an appropriate knowledge translation model to be used as a framework in the current study [11]. Support for using PARIHS as a frame can be found in Damschroder et al’s overview of theories in the knowledge translation field, where key constructs regarding implementation were found to include the PARIHS elements evidence, culture, leadership and evaluation [10]. This overview also support the additional categories identified in the analyses of the interviews in our pilot, i.e. organizational structure and the rehabilitation process related to patient needs, and resources [10]. Thus, we suggest that using PARIHS as an implementation study framework is feasible and relevant.

### Surveying rehabilitation interventions provided at the units

The subgroup of 7 patients who participated in the interviews and observations were identified by staff, which is a limitation due to the risk of bias. Instead, in a full scale study, to prevent bias, eligible patients should be identified through health care records, following a standardized procedure performed by trained data collectors. The interviews with patients were found to be acceptable and provided information on the patients’ perception of the rehabilitation interventions, but were not a feasible tool to survey the rehabilitation interventions provided. Instead it was feasible to identify these interventions through the notes in the health care records. The notes from the entire rehabilitation period also provided information on whether the patients were receiving interventions according to the 3 guideline recommendations. Moreover, the type of interventions provided, the number of visits, and the duration of the rehabilitation period could also be identified from the records. Retrospective data collections from health care records to survey adherence to clinical stroke guidelines is a common approach; the results in terms of such adherence have been found to vary [26].

One barrier to guideline use is that national guidelines are considered by practitioners not to be sufficiently concise and specific [27]. One example of a reported barrier to adoption is lack of information on frequency, duration, and intensity of mobility interventions [28]. In the present study the results from the assessment of data from the health care records indicated that the provision of out-patient care comprised rehabilitation interventions according to the SNGSC. However, as the outcomes of the interventions were not assessed in a standardized manner by the health practitioners, the sufficiency of the interventions described in the SNGSC cannot be fully evaluated. With a mean value of only 5 home visits per patient, and the financial restraints reported in the interviews that directed the outline of the interventions, the conditions for achieving a favorable patient outcome can be questioned. The SNGSC provides only a brief description of what the recommended interventions should entail, but defines the expected outcome of an intervention (e.g. as specified in Table 1). Thus, the assessment of outcome is crucial when evaluating adherence to guidelines such as the SNGSC. It is evident that managers and health practitioners will not know if the frequency, duration and intensity of an intervention provided are sufficient unless the presumed positive effects of an intervention are evaluated. Relevant, valid and structured assessments of patient outcome may provide valuable feed back to managers and health practitioners on the quality of the rehabilitation intervention and enhance the use of standardized updated clinical procedures.

### Use of standardized assessment tools

The assessment tools tested in this pilot were chosen for their suitability to assess patient outcomes after stroke rehabilitation in out-patient health care in accordance with the 3 recommendations in the SNGSC. The majority of these assessments were found to be feasible and acceptable to the patients when performed in the patients’ homes. However, the use of the Mini Mental Test Examination to assess cognitive impairments among patients receiving rehabilitation in out-patient care after stroke should be questioned. When compared to self-perceived cognitive ability in the Stroke Impact Scale, the results of the Mini Mental Test Examination indicate a ceiling effect. Instead the The Montreal Cognitive Assessment (MoCa) could be considered, as MoCa is more suitable for patients with milder cognitive impairments [29].

According to the results from using the Barthel Index to assess dependence in personal care and mobility [30], a ceiling effect was also indicated when compared to self-rated limitation in mobility and activities of daily living rated in the Stroke Impact Scale [31]. These findings are in line with results in a previous study [32]. Moreover, the use of Katz Extended ADL-Index to assess dependence in domestic life and transportation was not found to be feasible. Questions regarding dependence in ADL, in the Katz Extended, were described as hard to respond to by patients who did not perform the task before stroke while the questions in the Stroke Impact Scale regarding self-perceived experiences of limitations in ADL were less difficult to respond to. These results indicate that both Barthel Index and Katz Extended can be excluded in favour of the Stroke Impact Scale. These findings also point to a general need for testing assessment tools on the targeted patient group in the planning stage of an intervention study, as valid and reliable assessment tools can show floor or ceiling effects in different phases of recovery or progress of a disease.

## Conclusion

In this feasibility study a variety of qualitative and quantitative data collection procedures and measures were tested. The results indicate what can be used as a set of feasible and acceptable data collection procedures and measures suitable for studying implementation of stroke guidelines in an out-patient health care context. When planning a full scale study, procedures and measures to be considered are:

1. Using interviews with managers to survey the health care context at the units.
2. Minimizing the involvement of clinical staff in recruitment and data collection in favour of trained data collectors to ensure consistency and to ease the burden on clinical staff.
3. Identifying eligible patients from local hospitals rather than from out-patient units to survey the full reach of guideline use.
4. Using piloted measures of patients’ functioning and disability, assessed by trained data collectors, to evaluate results of guideline use.
5. Retrieving data from health care records to assess the health practitioners’ adherence to guidelines and their use of standardized assessments to evaluate patient outcome.

## Additional files

Additional file 1:
**Interview guide PRE staff.** (DOC 95 kb) Additional file 2:
**Interview guide POST staff.** (DOC 91 kb) Additional file 3:
**Interview guide PRE manager.** (DOC 96 kb) Additional file 4:
**Interview guide patient.** (DOC 87 kb)
