# Supplementary material for: Assessing feasibility and acceptability of study procedures: getting ready for implementation of national stroke guidelines in out-patient health care
Source: BMC Health Serv Res. 2015 Nov 23;15:517. doi: 10.1186/s12913-015-1177-5 (PMC4657360; doi:10.1186/s12913-015-1177-5)
Supplement: Additional file 1: — Interview guide PRE staff. (DOC 95 kb) [file 12913_2015_1177_MOESM1_ESM.doc]

**Interview guide – staff pre-intervention**

**Please tell me about:**

- your experience as a team member
- your background
- professional background

**With regards to this project – please tell me your views on:**

- the project

**Considering your work - please tell me:**

- about your role at the unit
- about your experience of stroke rehabilitation
- about your work with stroke rehabilitation in every day practice
- how you and your colleagues manage problems in everyday clinical practice

Regarding in house training –

- please tell about occurrence, provision and contribution to rehabilitation

Please tell me about other ways of learning things that you need in you every day work and how efficient you think they are

Please tell me your experience of what influences the outline of the rehabilitation interventions at your unit

Has conditions for providing rehabilitation changed over time

Please tell me:

- how new routines for providing and performing rehabilitation are developed and how implementation of new routines is facilitated and evaluated
- how you learn about new guidelines
- your experience of the application of guidelines in the clinical setting
- how rehabilitation interventions are evaluated
- if there are areas in rehabilitation where there is a disagreement between health professionals on what is ideal rehabilitation
- if you would like to change anything in particular regarding rehabilitation
- what you like best about your work
