# Supplementary material for: Assessing feasibility and acceptability of study procedures: getting ready for implementation of national stroke guidelines in out-patient health care
Source: BMC Health Serv Res. 2015 Nov 23;15:517. doi: 10.1186/s12913-015-1177-5 (PMC4657360; doi:10.1186/s12913-015-1177-5)
Supplement: Additional file 2: — Interview guide POST staff. (DOC 91 kb) [file 12913_2015_1177_MOESM2_ESM.doc]

**Interview guide - staff post intervention**

- **Please tell me:**
  - what you have come to know about this project
  - what your manager/s has/have done
  - which activities have affected you
  - the project’s influence on your everyday work
  - about things that have been difficult in the project
  - about things that have been easy in the project
  - about any changes since the project started*
  - how feed-back is provided*
  - your experience of the Swedish National Guidelines for Stroke Care
  - About the recruitment of patients to this project
- If you were told that a similar project was planned, what would you think
- Anything in particular that you have thought of/think of that you would like to share

*Refers to the intervention, addressed in a parallel paper
