# Supplementary material for: Assessing feasibility and acceptability of study procedures: getting ready for implementation of national stroke guidelines in out-patient health care
Source: BMC Health Serv Res. 2015 Nov 23;15:517. doi: 10.1186/s12913-015-1177-5 (PMC4657360; doi:10.1186/s12913-015-1177-5)
Supplement: Additional file 3: — Interview guide PRE manager. (DOC 96 kb) [file 12913_2015_1177_MOESM3_ESM.doc]

**Interview guide – managers pre intervention**

- **Please tell me about:**
  - your role as a manager
  - your background
  - your professional background
- **With regards to this project – please tell me your views on:**
  - the project
  - the intervention*
- **Considering your work, please tell me:**
  - what affects the outline of rehabilitation interventions provided at your unit
  - if the conditions for rehabilitation have changed
  - about changing the routines for rehabilitation
- about your role as a facilitator of change in the unit
- about new guidelines that are launched, how guidelines are applied in the clinical setting
- how rehabilitation interventions are evaluated
- if there are areas in rehabilitation where there is a disagreement between health professionals as to what is ideal rehabilitation
- if you would like to change anything in particular regarding rehabilitation
- what you like the best about your work

*Addressed in a parallel paper
