# Supplementary material for: Assessing feasibility and acceptability of study procedures: getting ready for implementation of national stroke guidelines in out-patient health care
Source: BMC Health Serv Res. 2015 Nov 23;15:517. doi: 10.1186/s12913-015-1177-5 (PMC4657360; doi:10.1186/s12913-015-1177-5)
Supplement: Additional file 4: — Interview guide patient. (DOC 87 kb) [file 12913_2015_1177_MOESM4_ESM.doc]

**Interview guide – patients**

- **Please tell me**
  - about your life situation in general (what is important, different, difficult in everyday life after stroke)
- **Regarding stroke rehabilitation, please tell me about**
  - your expectations on rehabilitation interventions after stroke
  - the interventions you have received
  - your participation in the rehabilitation and decision making, how you and the rehab staff discuss these issues
  - what is important to you in terms of your rehabilitation
- Is there anything you would have liked to change about your stroke rehabilitation?
- Is there anything in particular that you think of that you would like to share regarding these issues?
